# Regional default mode network connectivity in major depressive disorder: modulation by acute intravenous citalopram

**DOI:** 10.1038/s41398-019-0447-0

**Published:** 2019-03-15

**Authors:** Arpan Dutta, Shane McKie, Darragh Downey, Emma Thomas, Gabriella Juhasz, Danilo Arnone, Rebecca Elliott, Steve Williams, J. F. William Deakin, Ian M. Anderson

**Affiliations:** 1 Neuroscience & Psychiatry Unit, Institute of Brain, Behaviour and Mental Health and Manchester Academic Health Sciences Centre, Stopford Building, University of Manchester, Manchester, M13 9PT UK; 2 MTA-SE-NAP B Genetic Brain Imaging Migraine Research Group, Hungarian Academy of Sciences, Semmelweis University, Budapest, Hungary; 3 Department of Pharmacodynamics, Faculty of Pharmacy, Semmelweis University, Budapest, Hungary; 4 Centre for Affective Disorders, Institute of Psychiatry, Psychology and Neuroscience, King’s College London, De Crespigny Park, London, SE5 8AF UK; 5 Centre for Imaging Science and Manchester Academic Health Sciences Centre, Stopford Building, University of Manchester, Manchester, M13 9PT UK

## Abstract

The relationship between altered default mode network (DMN) connectivity and abnormal serotonin function in major depressive disorder (MDD) has not been investigated. Using intravenous citalopram and resting-state fMRI, we investigated DMN intra-network connectivity and serotonin function in 77 healthy controls and patients with MDD. There were no significant main effects of MDD or citalopram on DMN intra-network connectivity; however, significant interactions indicated that group differences under saline were modified by citalopram. In MDD patients during saline infusion, in contrast with controls, the DMN (i) did not include the precuneus that was instead part of an anti-correlated network but (ii) did include amygdala that was part of the anti-correlated network in controls. Citalopram infusion in MDD patients restored the pattern seen in controls under saline. In healthy controls, citalopram infusion disengaged the precuneus from the DMN and engaged the amygdala, partially reproducing the abnormalities seen under saline in MDD. In exploratory analyses within the MDD group, greater rumination self-ratings were associated with greater intra-network connectivity of the anterior cingulate cortex with the DMN. We hypothesise that, in MDD, disengagement of the precuneus from the DMN relates to overgeneral memory bias in rumination. The opposite effect, with greater engagement of the amygdala in the DMN, reflects the negative valence of rumination. Reversal of these abnormalities by citalopram suggests that they may be related to impaired serotonin function. That citalopram engaged the amygdala in the DMN in controls may relate to the paradoxical effects on aversive processing seen with acute SSRIs in healthy subjects.

## Introduction

Structural and functional magnetic resonance imaging (fMRI) studies have implicated abnormal anterior cingulate cortex (ACC) structure and function in the pathogenesis of depression and as a site of action of antidepressant action^1–5^. We recently found that ketamine evoked blood-oxygen-level-dependent (BOLD) responses in ACC that predicted improvement in mood 1 and 7 days later in major depressive disorder (MDD) patients^6^. However, the ACC does not function in isolation and it is a component in several neural systems relevant to depression^7,8^.

Functional networks involving ACC have been inferred from correlated patterns in the slow oscillations of BOLD signal detected in resting-state fMRI (rsfMRI). A resting-state network consists of a set of regions whose BOLD fluctuations inter-correlate^9,10^. The network can be defined by an abstracted BOLD waveform (independent component) with which the regional BOLD fluctuations correlate (independent components analysis (ICA))^11^. The degree to which regions correlate with the independent component is taken as a measure of their connectivity or integration with the whole network—intra-network connectivity^12^. Direct connectivity between components of a network are often assessed using regional correlations with the waveform from one or several seed regions to define the network^13,14^. The ACC is prominent in the default mode network (DMN), which includes posterior cingulate cortex (PCC; often used as a seed region), precuneus, medial prefrontal cortex (mPFC), ventral ACC and lateral and inferior parietal cortex^15^.

Several lines of evidence suggest that the DMN is involved in self-reflection, autobiographical memory and social cognition. The posterior cingulate/precuneus is one of the most metabolically active regions of the brain at rest and may be particularly important in self-reflective thinking^16,17^ in view of its role in visual imagery^18^ associated with autobiographical memory. The self-reflective role of the DMN has led to much interest in the possibility that excessive rumination in depression involves excessive DMN activity and an inability to switch out of it in response to external demands^19^. Rumination involves excessive negative inner preoccupation about the personal past, present and future. There is evidence that a tendency to ruminate is a personality trait that predisposes to depression and depressive relapse^20,21^. However, few studies have related DMN changes to questionnaire measures of rumination, the most widely used of which is the Ruminative Response Scale (RRS)^22^. RRS subscales cover negative thoughts associated with depression: brooding rumination and reflective rumination, of which the last may be a constructive form of rumination that is associated with better outcomes^23^. One study reported that dominance of the DMN over the task positive network (TPN) correlated with brooding rumination but not with reflective rumination; however, DMN/TPN dominance was no greater in MDD patients than in controls^23^. In an ICA study, Zhu et al.^24^ reported rumination scores correlated with greater ACC-DMN connectivity while impaired PCC-DMN connectivity correlated more with overgeneral memory recall in MDD patients. Seed-based studies found PCC–subgenual cingulate (SgACC) and SgACC–medial frontal cortex connectivity correlated with RRS scores^25,26^. An earlier literature found that high ratings of trait rumination were associated with prolonged amygdala responses to aversive stimuli^27,28^. One aim of the present study was to determine whether citalopram-induced 5-HT release modulates brain circuits associated with rumination as predicted by some theories of 5-HT and resilience^29^.

Several studies have investigated whether excessive DMN connectivity and other measures of DMN function are associated with MDD but many different methods have been employed and the results are inconsistent. In our review of fMRI resting-state networks, 13 studies reported increased resting-state activity or connectivity of the PCC/precuneus in MDD but 10 reported no change^11^. In a review of more recent ICA and seed-based studies, Mulders et al.^30^ conclude that a number of studies are compatible with an increase in connectivity within the anterior DMN connectivity (most consistently, the sgACC and mPFC) but that connectivity with the posterior network shows a variety of changes in MDD.

The present analysis sought to determine whether acute modulation of serotonin function using intravenous (i.v.) citalopram would modify ACC-DMN abnormalities in depression that are implicated in mechanisms of antidepressant action. We hypothesised that networks associated with the ACC would be abnormal in depression and associated with rumination and that acute i.v. citalopram challenge would normalise the abnormality.

## Materials and methods

### Subjects

Two studies, Remission Mechanisms in Depression (REMEDi) and New Molecules for Mood Disorders (NewMood) were performed at the University of Manchester in accordance with UK ethical procedures and the Declaration of Helsinki, with participants recruited via advertisement. The participants had no previous treatment with a selective serotonin reuptake inhibitor (SSRI) and were unmedicated.

The effects of acute citalopram infusion in currently depressed unmedicated MDD and non-MDD control participants were compared using data from the REMEDi study in which participants were randomised to receive 7.5 mg citalopram or saline i.v. over 7.5 min. Thirty-six unmedicated MDD participants were recruited to REMEDi (24 citalopram, 12 saline) together with 24 never-depressed (non-MDD) participants (13 citalopram, 11 saline). Non-MDD numbers were augmented using 14 participants from the NewMood study all of whom had received 7.5 mg citalopram i.v. in an identical imaging and infusion protocol to REMEDi, carried out in the same scanner by the same research group. Thus a total of 51 participants received citalopram (24 MDD, 27 non-MDD) and 23 received saline infusion (12 MDD, 11 non-MDD).

Participants were assessed using the Structured Interview for Diagnostic and Statistical Manual of Mental Disorders, Fourth Edition (DSM-IV)^31^. Patients met criteria for a DSM-IV major depressive episode. Healthy controls were required to have no personal or family history of psychiatric disorder. Patients’ illness severity was established using the Montgomery Åsberg Depression Rating Scale (MADRS)^32^ and the seven item Clinical Anxiety Scale adapted from the Hamilton Anxiety Rating Scale^33^. Currently depressed patients were required to have a MADRS score of ≥20 and psychotropic drug free for at least 2 weeks. The RRS was completed by all participants to measure rumination prior to infusion^22^.

Exclusion criteria were: depressive episode >2 years, depression superimposed on dysthymia, failure to respond to adequate treatment trial of 2 antidepressants or to citalopram/escitalopram in the current episode, allergy or intolerance to citalopram or escitalopram, contraindications to SSRI treatment, electroconvulsive therapy or lithium treatment in the current episode, significant suicidal risk, concurrent psychotropic medications, co-morbid Axis 1 psychiatric disorders except anxiety disorders (those with obsessive compulsive disorder were excluded), or personality disorder. Also excluded were participants with any unstable medical condition, pregnancy, neurological disorders, history of significant head trauma, lifetime history of substance or alcohol abuse, current alcohol use over 14 units for women or 21 units for men (these were UK government recommendations at the time the data were collected), caffeine usage >6 cups of coffee per day, smoking >10 cigarettes per day and any contraindication to MRI scanning. For the REMEDi study, the research pharmacy generated and held the randomisation codes and dispensed the citalopram or saline on the day of the experiment. The infusion was made up by an independent research nurse and labelled only with the participant code to maintain the blindness of the research team. Informed consent was obtained from all participants. Ethical approval was gained from the local research ethics committee before each study.

### MRI acquisition and preprocessing

Images were acquired on a Philips Intera 1.5 Tesla MR scanner housed within the NIHR/Wellcome Trust Clinical Research Facility, Manchester, UK. The acquisition parameters were single-shot echo-planar imaging (EPI) sequence with an ascending and sequential slice order to give full brain coverage with isotropic (3.5 mm) voxels (35 slices, 0.5 mm slice gap 64 × 64 matrix, TE (echo time) = 40 ms, TR (time to repetition) = 2 s). Participants were asked to lie still. Total scan duration was 25 min during which citalopram 7.5 mg or matching saline were infused i.v. over 7.5 min after a 5-min baseline period. Scanning was continued for a further 12½ min and this second half of the scan following infusion was used for analysis. A qualified radiographer performed all MR scans. Images were preprocessed using Statistical Parametric Mapping (SPM)^34^ run under MATLAB 2013a^35^.

Images were realigned using the first volume as a reference and a six-parameter rigid body transformation. Co-registration of the structural image and EPI images from realignment was performed. This was followed by segmentation of the structural image into grey and white matter. The grey matter segmented image was normalised to the Montreal Neurological Institute (MNI) grey matter probability map supplied with SPM. The normalisation transform matrix was applied to the realigned functional images. The normalised functional images were then smoothed using a 10-mm full-width half maximum isotopic Gaussian kernel.

Resting-state analysis connectivity analysis has been shown to be sensitive to being confounded by head motion due to global intensity changes^36–38^. To address this, we examined two motion parameters suggested by Power et al.^36^: framewise displacement (FD) and DVARS (the temporal derivative of timecourses RMS variance of the differentiated timecourse over each voxel for every volume). FD measures the movement of any frame relative to the previous frame. DVARS quantifies volume-to-volume BOLD signal change (See supplementary material tables).

### Group-independent component analysis

ICA is a data-driven model-free method^39^ of identifying networks of brain regions that show inter-correlated slow fluctuations in resting-state BOLD signals. ICA imputes independent BOLD signal timecourses (independent components (ICs)) that account for patterns of inter-correlation. The preprocessed images were entered into Group ICA of fMRI Toolbox (GIFT) software run under MATLAB 2013a^40^. The group ICA produces a map of the correlation between regional BOLD signals and each of 20 ICs based on the mean data length. The regional correlations with the independent component are considered to be measures of connectivity showing a region’s connectivity with the network or ‘intra-network connectivity’. The IC map with greatest spatial correlation with an ACC template (Wake Forest University PickAtlas)^41–44^ was objectively selected as the ACC-containing network using a multiple regression model. The independent component with the strongest anatomical correlation with the ACC mask proved to be the DMN identified in the GIFT toolbox; thus ‘ACC-containing network’ and DMN are one and the same in this study. The group ICA also identifies regional BOLD responses that are anti-correlated with the IC, in this case, anti-correlated with the DMN component. From the group ICA, individual maps of regional correlation with the DMN (ACC-containing network) were back-reconstructed using GIFT. Parametric cluster-forming thresholds and family-wise error correction at the cluster level leads to an unacceptable amount of type 1 errors^45^. Therefore, the analysis was performed using non-parametric statistical tests using Multivariate and repeated measures (MRM) toolbox^46^. MRM is a MATLAB-based toolbox used to analyse group models of neuroimaging data using the multivariate general linear model. The intra-network connectivity maps of the ACC-containing network from each individual were entered into a 2 × 2 factorial model with group (MDD vs control) and treatment (citalopram vs saline infusion) as between-subject factors.The multivariate test statistic used was Pillai’s trace. Family-wise error cluster level inference (pFWEc) was used at *p* < 0.05 with a cluster-forming height threshold of *p* = 0.002 (two tailed) was used for all analyses with the number of permutations set at 10,000. In subsidiary analyses, MADRS depression ratings and RRS total score and subscale ratings were entered separately as covariates in the complete model and also in the MDD group alone.

### Depression and rumination scores

Demographic data, MADRS and RRS total and subscale scores were analysed using two sample *t* tests to evaluate group differences.

## Results

### Participant characteristics

Table 1 shows the study participant characteristics. There were no significant differences in mean age between the groups although healthy controls were slightly younger than MDD participants. There were more females than males in all groups, with no statistically significant differences in sex distribution among the groups. Only the healthy control citalopram group was augmented and there was no statistically significant difference between the NewMood and REMEDi healthy controls administered citalopram. The baseline MADRS scores were significantly greater in the MDD groups compared to the healthy controls groups and comparable in those patients receiving citalopram or saline. Mean FD was 0.29 and mean DVARS 0.71. There was an effect of group but not treatment in the motion parameters.

**Table 1:** Participant characteristics and mood rating scales

	Healthy controls		Current MDD	
	Citalopram	Saline	Citalopram	Saline
Sample size	27	11	24	12
Mean age, years	30.9 (SD 9.3)	32.7 (SD 8.6)	35.9 (SD 8.5)	36 (SD 10)
Males	7 (26%)	3 (27%)	8 (33%)	3 (25%)
Females	20 (74%)	8 (73%)	16 (67%)	9 (75%)
Baseline MADRS	0.6 (SD 1.3)^a^	0.0 (SD 0.3)^a^	26.8 (SD 4)	28 (SD 4.8)
RRS Total Score	37.3 (SD 11.3)	36.3 (SD 11.7)	60.2 (SD 9.3)	61.8 (SD 10.2)
RRS—depression	1.58 (SD 0.43)	1.56 (SD 0.61)	2.68 (SD 0.57)	2.75 (SD 0.60)
RRS—reflection	1.79 (SD 0.76)	1.82 (SD 0.66)	2.53 (SD 0.67)	2.63 (SD 0.62)
RRS—brooding	1.76 (SD 0.52)	1.62 (SD 0.56)	2.77 (SD 0.48)	2.72 (SD 0.60)

*MDD* major depressive disorder, *MADRS* Montgomery Åsberg Depression Rating Scale, *RSS* Ruminative Response Scale

^a^*p* < 0.05 for either healthy control group compared to either current MDD group

### Group-independent component analysis

**Fig. 1:**
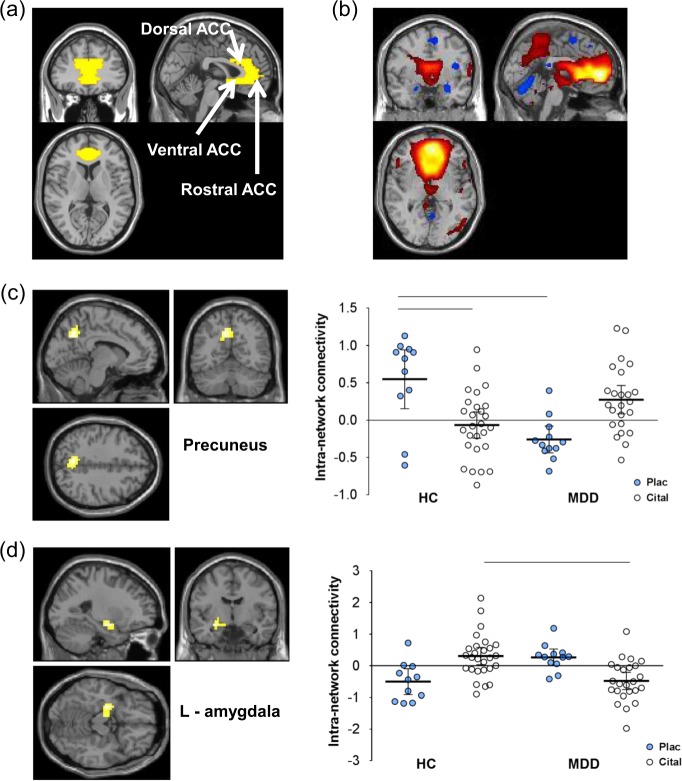
**Effects of citalopram vs placebo on intra-network connectivity**. **a** Anterior cingulate cortex (ACC) spatial correlation template. **b** ACC resting-state network map—effects are plotted at peak-level threshold of *p* = 0.002 (red/orange = positive; blue = negative. **c**, **d** Scattergrams+Statistical Parametric Mapping contrast maps for the regions of ACC resting-state component that show a significant drug×treatment group interaction—effects are plotted at peak-level threshold of *p* = 0.002 and extent threshold of *p*(FWEc) < 0.05 with 95% confidence intervals. HC healthy controls, MDD major depressive disorder, cital citalopram, plac placebo; pairwise comparisons at *p*(FWEc) < 0.05 indicated by horizontal lines, see supplementary material

Figure 1b shows the mean intra-network connectivity map for the ACC-containing network for all participants. The spatial correlation to the ACC template (Fig. 1a) was *r* = 0.454. Apart from ACC, the regions showing significant intra-network connectivity included most elements of the DMN, including posterior cingulate, precuneus and angular gyrus (supplementary material Fig. 1 and Table 3). In addition, GIFT identified an anti-correlated network involving right and left anterior and medial temporal regions, including amygdala and parahippocampal gyrus (Figure 1 supplementary material).

There were no significant main effects of diagnosis or drug on intra-network connectivity in any region of the ACC network. Statistically significant drug×diagnosis interactions were found in the left precuneus and left amygdala indicating that acute citalopram had different effects in the two groups (Table 2). In healthy controls under saline, left precuneus showed strong positive connectivity with the ACC (default mode) network. MDD patients in contrast showed mean negative connectivity (anti-correlation) with the ACC network with the 95% confidence intervals almost completely below zero (Fig. 1c). However, during i.v. citalopram infusion MDD patients showed positive connectivity similar to untreated controls. Citalopram infusion in controls was associated with a non-significant reduction in ACC connectivity compared with saline-infused controls.

**Table 2:** Drug×group interaction

Region	Side	Co-ordinates			No. of voxels	*p*(FWEc)
		*x*	*y*	*z*		
Amygdala	L	−26	−7	−15	27	0.001
Precuneus	L	−5	−63	40	41	0.001

*p(FWEc)* family-wise error-corrected *p* value at the cluster level, *L* left

Amygdala BOLD signal under saline in healthy controls was anti-correlated (negative correlation) with the ACC network, whereas in MDD patients, amygdala showed positive connectivity (positive correlations) with the DMN (Fig. 1d). During citalopram infusion, this pattern was reversed; in controls amygdala BOLD signal was positively correlated with the ACC network, whereas in MDD patients amygdala was anti-correlated with the ACC network component as in the saline-treated controls.

### Rating-scale correlations with intra-network connectivity

**Fig. 2:**
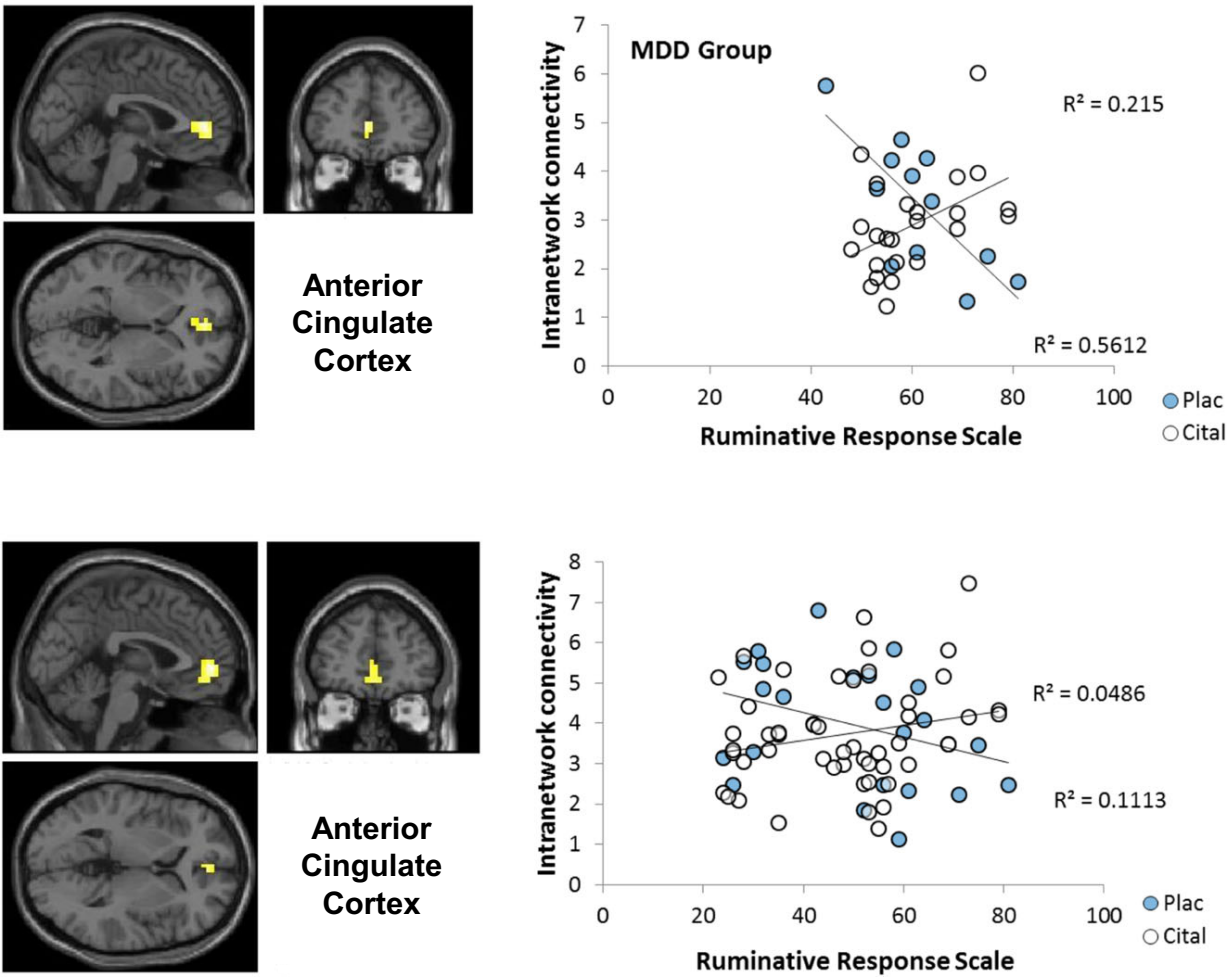
Statistical Parametric Mapping contrast maps and scatterplots of regions that correlated with Ruminative Response Scale subscale scores. ACC correlation with RRS score (cital citalopram, plac placebo)

Severity of depression as measured by MADRS scores did not correlate with intra-network connectivity of any region (in keeping with the lack of a main effect of group) or in any subgroup defined by interaction with group and/or drug. In contrast to the MADRS, total RRS scores correlated with intra-network connectivity of the rostral ACC in the MDD group but not in controls (Fig. 2a) producing a significant RRS-by-group interaction (Table 3).

**Table 3:** Regions that correlated with Ruminative Response Scale scores

Region	Side	Co-ordinates			No. of voxels	*p*(FWEc)
		*x*	*y*	*z*		
Ruminative Response Scale total						
MDD citalopram>saline						
Anterior cingulate (BA32)	L	−1	49	0	8	0.031
Citalopram>saline						
Anterior cingulate (BA32)	L	−1	53	0	20	0.008

*MDD* major depressive disorder, *L* left, *p(FWEc)* family-wise-error-corrected *p* value at the cluster level

Within the MDD group, ACC-DMN connectivity showed a significant total RRS-by-drug interaction (Fig. 2a; Table 3). Under saline, greater RRS scores were associated with reduced network connectivity; during citalopram infusion, greater DMN network connectivity was associated with increased RRS scores. RRS brooding, depression and reflective subscales were >0.63 in all participants and within the MDD group and are therefore not discussed further.

## Discussion

The key findings of the study are (i) that controls and MDD participants differed in how two key structures, implicated in mechanisms of rumination, are integrated with the DMN, and (ii) that the inference that these changes were modulated by acute citalopram-induced 5-HT release. We consider first the effect of MDD in saline-infused participants and second the effect of citalopram. In left precuneus, BOLD fluctuations in saline-treated controls were, as expected, correlated with the DMN network but they were anti-correlated in the MDD patients. In other words, the precuneus was not merely dissociated from the DMN in the depressed patients but instead showed aberrant connectivity with the anti-correlated network. In contrast, the amygdala, part of the anti-correlated network in controls, was integrated with the DMN in MDD participants (Fig. 1d). It must be noted that given the low power of the control groups these are preliminary data. Given the preliminary nature of the results, it is difficult to comment on whether similar results would have been observed using clinically effective dosing of oral citalopram.

The anti-correlated network to the DMN in the overall group ICA is summarised in supplementary material. The regions do not correspond to a single classically identified network but rather appear to involve elements of several networks; for example uncus and parahippocampal gyrus from the memory network, bilateral insula from the salience network, superior frontal gyrus from the task positive network and indeed parts of precuneus and angular gyrus associated with the DMN itself. The anti-correlated network may reflect the phasic suppression of components of other networks, possibly by top–down control from rostral ACC^47–49^ and the salience network in phases when the DMN is active^23^.

An emerging concept is that DMN-mediated self-reflection becomes maladaptive when other networks become integrated with the DMN. For example, Sheline et al.^7^ reported that in MDD the DMN and emotion networks overlap in a dorsomedial frontal cortical ‘nexus’ in a seed-based study. Hamilton et al.’s^50^ review and synthesis proposed that aberrant connectivity with an overactive SgACC mediates the affective tone of DMN self-reflection in MDD. The intrusion of left amygdala into the DMN IC in our MDD group is compatible with these ideas and could be a route by which rumination acquires negative emotional valence and becomes maladaptive. Other ICA studies have not reported amygdala correlation with the DMN in MDD. However, the masks used in several studies may have excluded the possibility of detecting such aberrant correlations. A study using bilateral amygdala seeds made the relevant observation of positive connectivity between amygdala and left precuneus in MDD compared to their negative connectivity seen in controls^51^. This suggests that abnormal amygdala–precuneus connectivity specifically could mediate the negative emotional valency of memory imagery in MDD. In our study, left amygdala and precuneus correlated with opposed DMNs in the healthy controls in keeping with Cullen et al.^51^; that they switched networks in the MDD group does not imply that the entirety of precuneus and amygdala anatomy and function remained segregated in different networks in our MDD group. Rather, the crossing-over of parts of amygdala and precuneus into anti-correlated networks probably represents a flexible merging of self-reflection and of emotion-processing elements of different networks in MDD. The functional significance of amygdala correlations with the DMN in our MDD group, however, remains unclear, since there was no correlation with depressive symptoms or with RRS scores.

In contrast with the amygdala–DMN changes, the shift in precuneus correlation away from DMN to the anti-correlated network in our MDD group correlated with greater RRS scores. Three other studies have reported correlation between RRS scores and RS connectivity but they are not immediately germane to our findings because they probed connections other than precuneus (see Introduction). A fourth study by Zhu et al.^24^ found decreased precuneus–DMN connectivity in MDD, but this correlated with an overgeneral memory recall measure rather than their measure of rumination, the 4-item rumination subscale from the Cognitive Emotion Regulation Questionnaire (CERQ-R)^52^. The CERQ-R scale focusses on cognitive responses to life events and differs considerably from the RRS and its subscales. Given the importance of rumination as a mechanism of overgeneral memory recall^53^, Zhu et al.’s findings on the functional correlates of precuneus–DMN disconnection may not be far removed from our own. A recent experiment attests to the importance of precuneus in overgeneral recall in depression. MDD patients were able to recruit the DMN in constructing experimental autobiographical memories but their impaired performance related to impaired activation of precuneus and of its seed-based connectivity with hippocampus^54^. Since some precuneus connectivity was shifted to the anti-correlated DMN in our MDD group rather than merely dissociated from the DMN, we speculate that it may be directly involved in repetitive rehearsal of widely represented depressive semantic self-knowledge, i.e. rumination, that interferes with specific recall of autobiographical memories.

Following our observations on the role of rostral ACC in mediating the antidepressant effects of acute ketamine infusion^6^, one motivation for the present analysis was to test whether ACC connectivity abnormalities in MDD would be affected by a single dose of an SSRI. However, we found no group differences in ACC-DMN correlation and no main effects of drug or drug-by-group interaction. Several studies have reported increased ACC DMN connectivity in MDD^30^, but this is a large region and it is not clear that a particular ACC subregion is consistently implicated except perhaps sgACC^55^. We observed a group interaction with rumination such that lesser rostral (pregenual) ACC DMN connectivity was correlated with RRS scores in MDD as found in precuneus but without evidence of association with the anti-correlated network or of modulation by citalopram. A possible inference is that MDD may involve some loss of ACC control of resting-state modes that might contribute to the intrusion of amygdala into the DMN and the dissociation of precuneus. However, caution is required since our finding is in apparent conflict with Zhu et al.’s observation that increased rather than decreased rostral ACC DMN connectivity in MDD correlated with their CERQ measure of rumination.

Citalopram infusion appeared to normalise the abnormalities of precuneus and amygdala integration with the DMN seen under saline infusion: the precuneus was integrated with the DMN and amygdala was anti-correlated as in saline-infused controls. One interpretation of the findings is that the loss of precuneus integration with the DMN and intrusion of the amygdala reflects an impairment of 5-HT neurotransmission in depression that is corrected by i.v. citalopram-induced 5-HT release. A difficulty for this idea is that citalopram had the opposite effects in the healthy controls in whom citalopram shifted an amygdala cluster towards the DMN and shifted out a precuneus cluster (albeit non-significantly) thus mimicking the effect of MDD on the DMN. A possible explanation is the recognised effect of acute SSRIs to induce anxiety variously attributed to stimulation of 5-HT_2C_ receptors^29,56^ or to a deficit of synaptic 5-HT through stimulation of cell-body auto-receptors^57^. More generally, the engagement of the amygdala in the DMN by citalopram in controls may be another example of many paradoxical findings that acute SSRI treatment enhances aversive processing and symptoms in animals and humans yet work in depression by increasing serotonin function.

This study has several limitations. The smaller numbers of saline- than citalopram-infused participants may have reduced the power to detect regions with hypothesised increase in DMN connectivity. A cross-over design was not considered practical but would have provided more power to detect group and drug effects. No filtering was applied to the data set to compensate for physiological noise contributing to increased variance in the data; however, this should have been removed by the spatial ICA. In addition, our analyses showed a potential effect of head movement in the depressed group compared to healthy controls regardless of treatment. Susceptibility artefact may have prevented replication of altered sgACC connectivity in MDD^50^. There are no other studies of acute 5-HT modulation of the DMN, so the findings are preliminary, although Klaassens et al.^58^ reported comparable effects of oral sertraline on precuneus connectivity in a single-dose study in 12 volunteers. Given the preliminary nature of the results, it is difficult to comment on whether similar results would have been observed using clinically effective dosing of oral citalopram.

We conclude that the DMN is likely to be abnormally configured in MDD with intrusion of amygdala connectivity and loss of some precuneus connectivity, rather than showing a general increase in connectivity; the quality of self-reflection rather than the amount may be the more important change in depression. The DMN abnormalities in MDD may involve altered serotonin modulation of connectivity because they were not seen during acute serotonin release evoked by i.v. citalopram. DMN connectivity in healthy volunteers and patient groups appears to be sensitive to experimental drug challenge and this could be an important approach to understanding neurotransmitter function in depression and in detecting effective new drugs.

## Supplementary information

Supplementary Material

Supplementary material
