# Supplementary material for: Regional default mode network connectivity in major depressive disorder: modulation by acute intravenous citalopram
Source: Transl Psychiatry. 2019 Mar 15;9:116. doi: 10.1038/s41398-019-0447-0 (PMC6420575; doi:10.1038/s41398-019-0447-0)
Supplement: Supplementary file 1 — Supplementary Material [file 41398_2019_447_MOESM1_ESM.pdf]

Supplementary material

Default mode network array

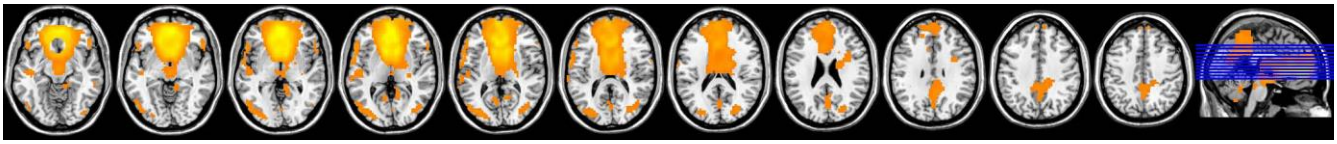

Default mode anti-correlated network

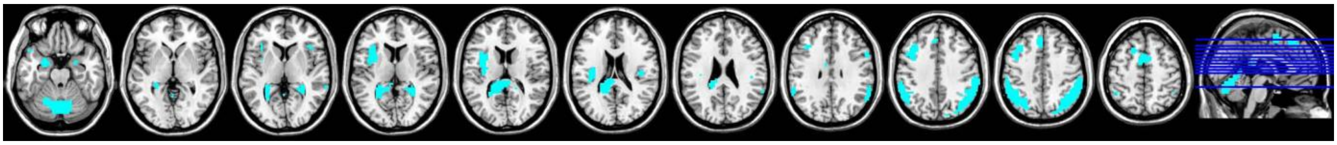

Figure 1 Independent component arrays from GIFT toolbox

Table 3 Regions correlated with the DMN

Area	Brodmann Area	Volume (cc)	Random effects: Max Value (x, y, z)
Anterior Cingulate	10, 24, 25, 32	5.4/5.2	22.1 (-1, 47, -2)/22.4 (2, 44, -2)
Right Cerebrum		1.2/1.7	9.9 (-1, 61, -3)/21.3 (2, 47, -7)
Extra-Nuclear		4.9/5.6	20.0 (-4, 28, 8)/18.0 (6, 31, 3)
Caudate		1.9/2.6	19.8 (-8, 17, 4)/19.0 (9, 14, 4)
Medial Frontal Gyrus	9, 10, 11	5.2/4.7	19.5 (-4, 51, -3)/19.6 (2, 51, -3)
Sub-Gyral		4.9/5.0	18.3 (-8, 31, 8)/16.5 (13, 34, -2)
Lateral Ventricle		1.7/1.9	17.4 (-4, 14, 4)/17.2 (13, 24, -1)
Superior Frontal Gyrus	8, 9, 10, 11	4.0/3.0	13.7 (-15, 47, -11)/12.5 (20, 43, -15)
Middle Frontal Gyrus	6, 10, 11, 47	2.0/2.1	12.9 (-18, 44, -11)/11.9 (20, 37, -14)
Inferior Frontal Gyrus	45, 47	2.0/2.0	12.4 (-22, 33, -10)/9.0 (23, 30, -10)
Thalamus		0.7/1.2	8.0 (-8, -3, 9)/10.3 (6, -3, 9)
Lentiform Nucleus		0.8/2.0	9.5 (-15, 13, -5)/9.7 (16, 13, -5)
Subcallosal Gyrus	13, 34, 47	0.3/0.3	7.2 (-11, 6, -13)/8.2 (16, 16, -13)
Precuneus	7, 31	2.1/2.5	5.9 (-1, -45, 48)/6.3 (2, -48, 53)
Precentral Gyrus	6, 43, 44	0.8/0.2	6.3 (-60, 11, 9)/4.5 (41, -17, 61)
Inferior Temporal Gyrus	19, 20	0.2/0.3	6.2 (-50, -71, -1)/3.7 (51, -52, -14)
Middle Occipital Gyrus	18, 19, 37	2.2/0.6	6.2 (-32, -81, 13)/4.2 (34, -71, 17)
Superior Temporal Gyrus	22, 41, 42	1.6/0.0	6.1 (-60, 4, 4)/na (0, 0, 0)
Paracentral Lobule	5	0.5/1.2	5.2 (-1, -35, 52)/6.0 (2, -38, 53)
Middle Temporal Gyrus	19, 21, 37, 39	1.1/0.8	5.8 (-36, -77, 18)/4.9 (51, -8, -16)
Cingulate Gyrus	23, 31, 32	1.0/2.1	5.4 (-1, -42, 34)/5.7 (9, -43, 30)
Third Ventricle		0.3/0.1	5.7 (-1, -7, -8)/4.6 (2, -4, -8)
Cuneus		0.3/0.3	5.2 (-25, -84, 13)/4.3 (23, -77, 22)
Cerebellar Tonsil		0.6/1.2	4.8 (-15, -49, -35)/4.9 (20, -53, -39)
Posterior Cingulate		0.4/0.3	4.8 (-8, -61, 12)/4.9 (9, -54, 7)
Culmen		0.2/0.6	4.5 (-11, -45, -19)/4.8 (9, -44, 2)
Nodule		0.1/0.1	4.4 (-8, -56, -27)/4.6 (13, -52, -27)
Fastigium		0.1/0.1	4.3 (-8, -49, -19)/4.5 (9, -56, -22)
Inferior Occipital Gyrus		0.0/0.1	-999.0 (0, 0, 0)/4.1 (41, -82, -4)
Inferior Semi-Lunar Lobule		0.1/0.1	4.0 (-4, -63, -35)/3.5 (2, -60, -39)
Lingual Gyrus		0.1/0.1	3.8 (-22, -88, 4)/4.0 (13, -47, 2)
Postcentral Gyrus		0.1/0.0	3.8 (-60, -6, 14)/na (0, 0, 0)
Transverse Temporal Gyrus		0.1/0.0	3.7 (-50, -20, 10)/na(0, 0, 0)
Parahippocampal Gyrus	30	0.0/0.1	Na (0, 0, 0)/3.5 (13, -38, -2)

Figure 2 Independent components with correlation scores to DMN template

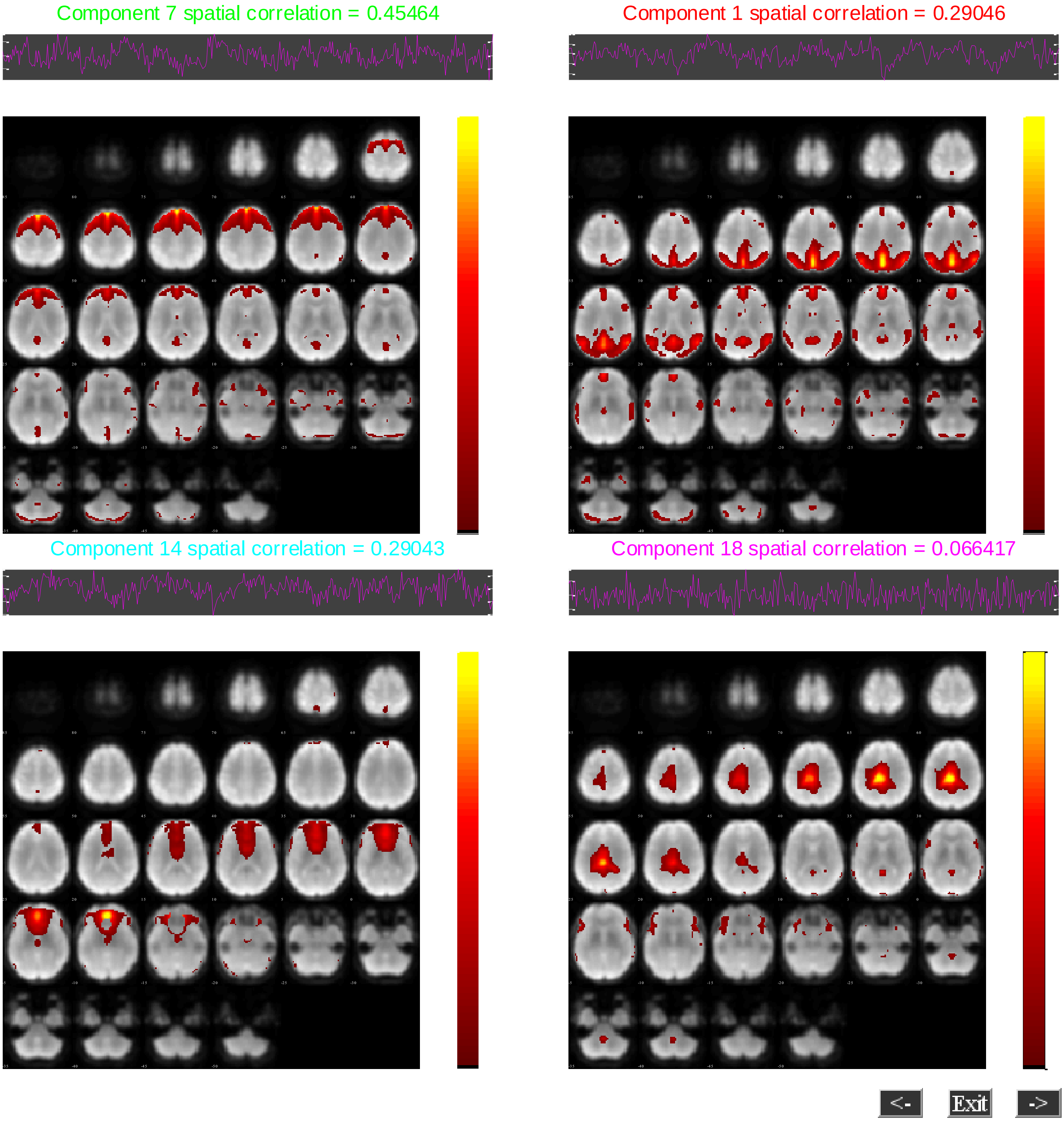

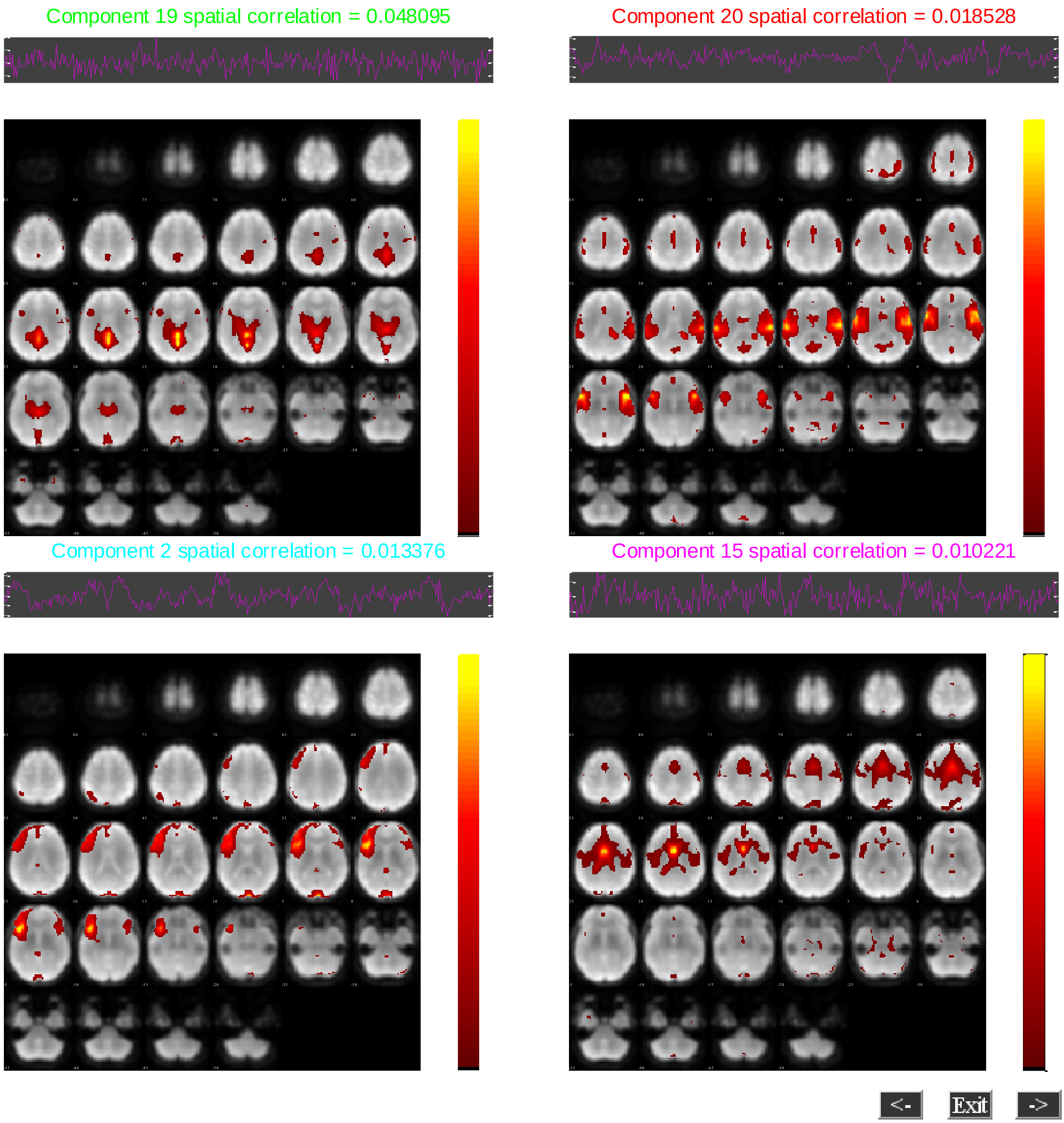
Component 19 spatial correlation = 0.048095
Component 20 spatial correlation = 0.018528
Component 2 spatial correlation = 0.013376
Component 15 spatial correlation = 0.010221
<-
Exit
->

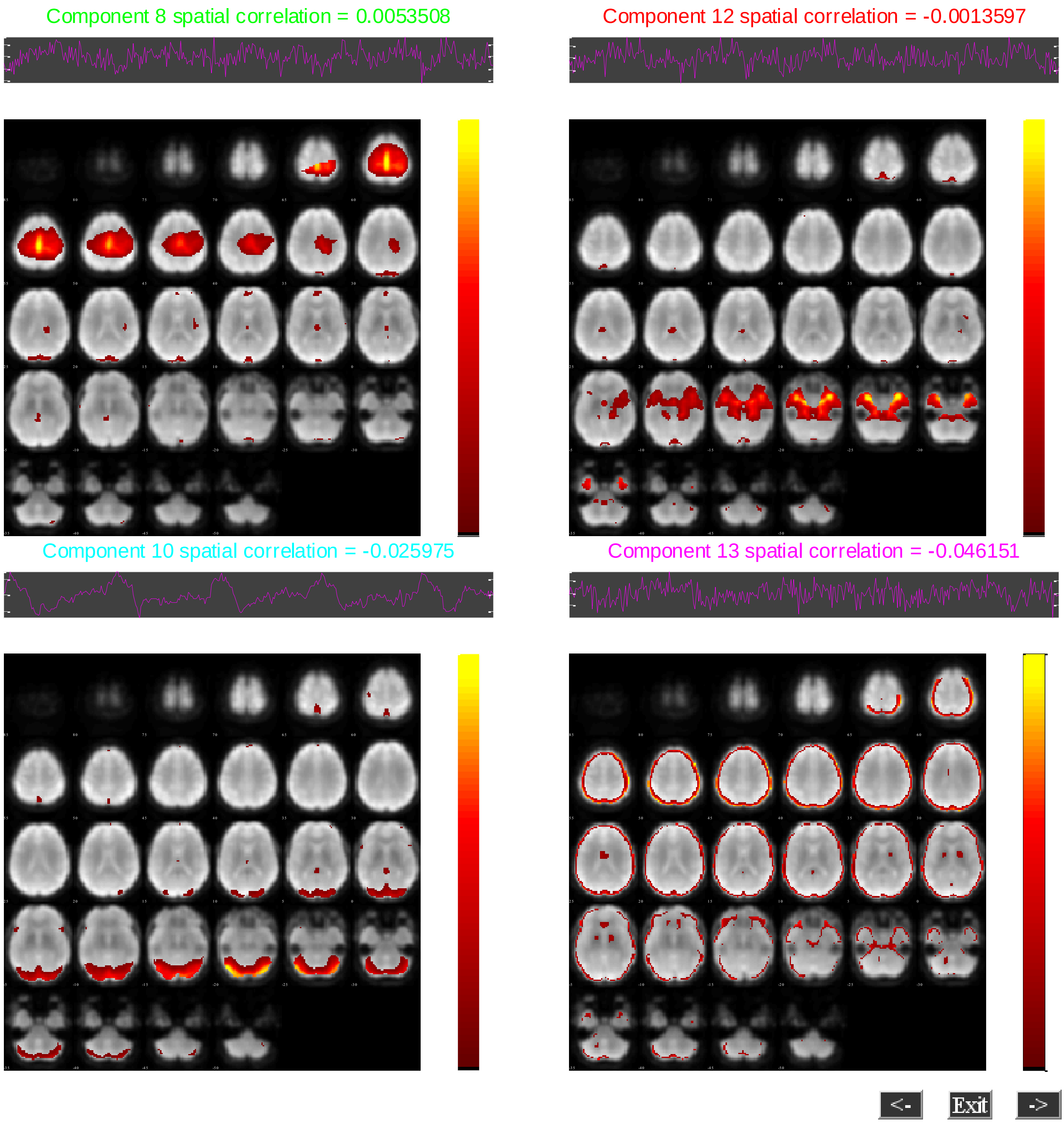
Component 8 spatial correlation = 0.0053508
Component 12 spatial correlation = -0.0013597
Component 10 spatial correlation = -0.025975
Component 13 spatial correlation = -0.046151
<-
Exit
->

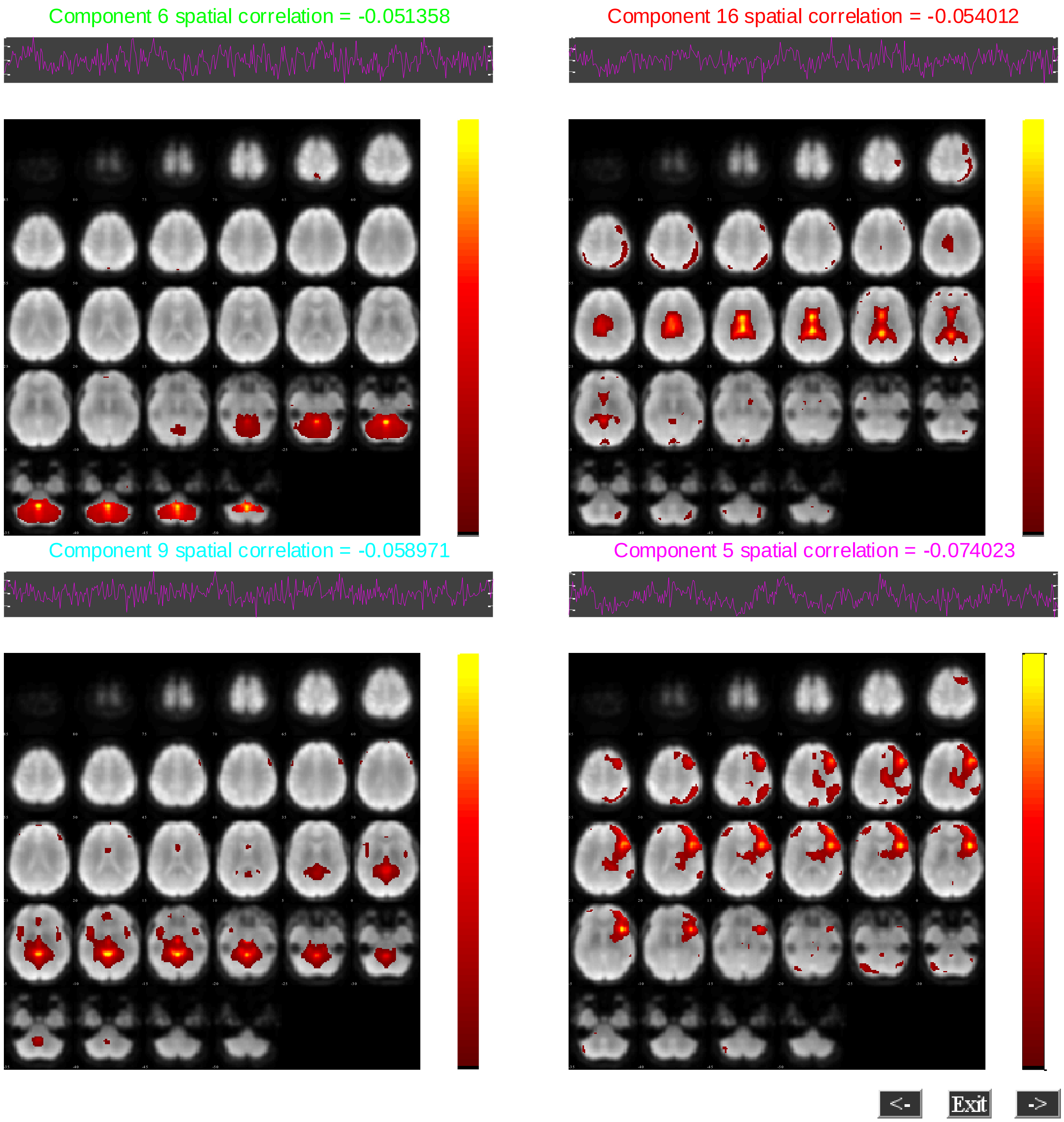
Component 6 spatial correlation = -0.051358
Component 16 spatial correlation = -0.054012
Component 9 spatial correlation = -0.058971
Component 5 spatial correlation = -0.074023
<-
Exit
->

Component 11 spatial correlation = -0.080777

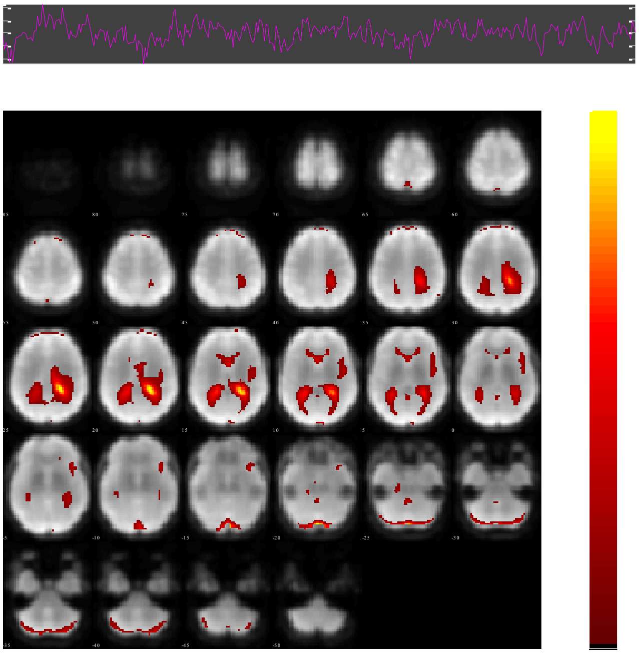

Component 4 spatial correlation = -0.087799

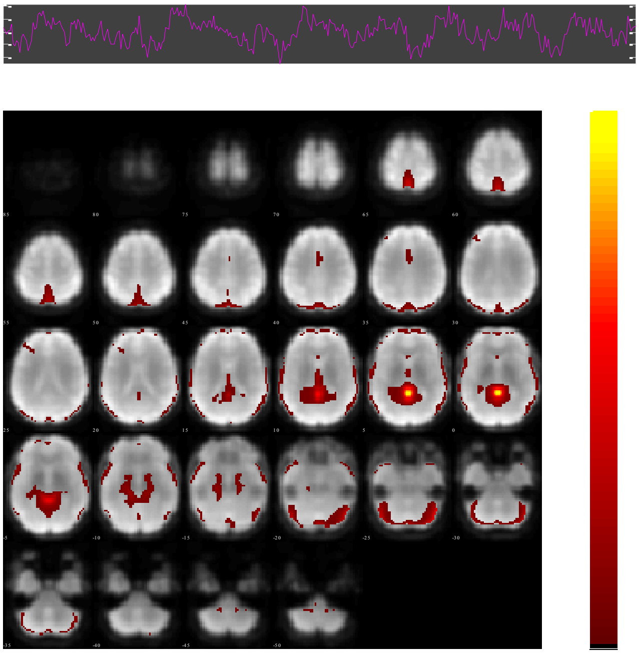

Component 17 spatial correlation = -0.091105

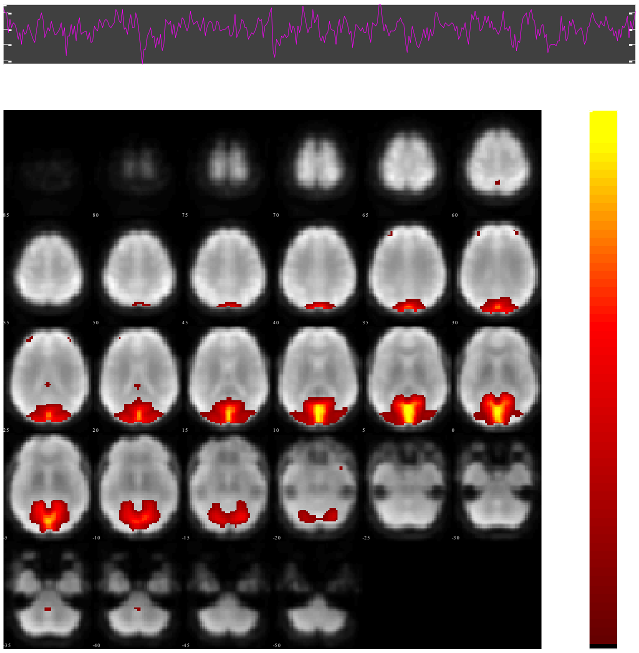

Component 3 spatial correlation = -0.11745

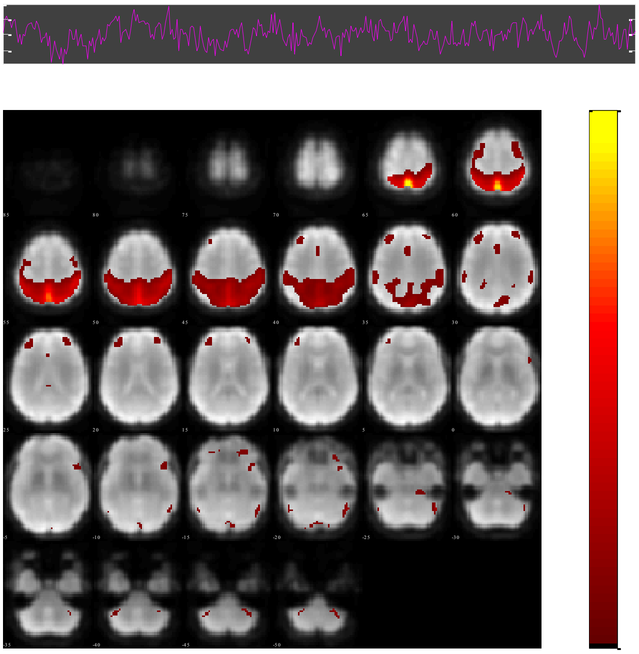

<- Exit ->
