# Supplementary material for: Regional default mode network connectivity in major depressive disorder: modulation by acute intravenous citalopram
Source: Transl Psychiatry. 2019 Mar 15;9:116. doi: 10.1038/s41398-019-0447-0 (PMC6420575; doi:10.1038/s41398-019-0447-0)
Supplement: Supplementary file 2 — Supplementary material [file 41398_2019_447_MOESM2_ESM.pdf]

Supplementary Material

Table 1 Post-hoc pairwise comparisons for group by drug interactions (fig 2c,d)

**Region**	**No of voxels**	**p(FWEc)**
**Small volume correction using interaction mask (precuneus)**		
Placebo(HC>MDD)	24	**0.004**
HC (placebo>citalopram)	8	**0.028**
**Small volume correction using interaction mask (amygdala)**		
Citalopram (HC>MDD)	10	**0.04**

p(FWE) = family wise error corrected p-value at cluster level

p values were multiplied by 4 within each mask group to correct for the four post hoc tests completed.

Table 2 Mean Frame Displacement (FD) and DVARS

**Mean FD**			
	HC	cMDD	
Citalopram	0.217614 (SD 0.08)	0.437562 (SD 0.26)	P<0.05 effect of group. No significant effect of treatment
Placebo	0.208711 (SD 0.07)	0.281471 (SD 0.12)	
**Mean DVARS**			
Citalopram	0.607666 (SD 0.11)	0.899369 (SD 0.30)	P<0.05 effect of group. No significant effect of treatment
Placebo	0.621146 (SD 0.08)	0.703382 (SD 0.20)	
